# Resistance exercise training-induced skeletal muscle strength provides protective effects on high-fat-diet-induced metabolic stress in mice

**DOI:** 10.1186/s42826-022-00145-0

**Published:** 2022-12-02

**Authors:** Hye Jin Kim, Youn Ju Kim, Il Yong Kim, Je Kyung Seong

**Affiliations:** 1 Laboratory of Developmental Biology and Genomics, College of Veterinary Medicine, Seoul National University, Seoul, 08826 Republic of Korea; 2 Korea Mouse Phenotyping Center (KMPC), Seoul National University, Seoul, 08826 Republic of Korea; 3 BK21 Program for Veterinary Science, College of Veterinary Medicine, Seoul National University, Seoul, 08826 Republic of Korea; 4 The Research Institute for Veterinary Science, College of Veterinary Medicine, Seoul National University, Seoul, 08826 Republic of Korea; 5 Interdisciplinary Program for Bioinformatics, Program for Cancer Biology, BIO-MAX/N-Bio Institute, Seoul National University, Seoul, 08826 Republic of Korea

**Keywords:** Resistance exercise, Skeletal muscle, Obesity, Insulin sensitivity, High-fat diet

## Abstract

**Background:**

Resistance exercise training is known to improve metabolic disorders, such as obesity and type2 diabetes. In this study, we investigated whether the beneficial effects of resistance exercise training persisted even after the discontinuation of training with high-fat diet (HFD)-induced metabolic stress. We further evaluated whether the improvement in skeletal muscle strength and endurance by training were correlated with improved metabolism. Eight-week-old male C57BL/6N mice were divided into groups that remained sedentary or had access to daily resistance exercise via ladder climbing for 8 weeks. Trained and untrained mice were fed an HFD for 1 week after the exercise training intervention (n = 5–8 per group).

**Results:**

Resistance exercise-trained mice had a lean phenotype and counteracted diet-induced obesity and glucose tolerance, even after exercise cessation. Grip strength was significantly inversely correlated with the body weight, fat mass, and glucose tolerance. However, hanging time was significantly inversely correlated with body weight only.

**Conclusions:**

These results have strong implications for the preventive effect of resistance exercise-induced metabolic improvement by enhancing skeletal muscle strength rather than endurance.

**Supplementary Information:**

The online version contains supplementary material available at 10.1186/s42826-022-00145-0.

## Background

Although the effects of exercise on metabolic outcomes differ according to age, gender, exercise type, intensity, and duration, exercise-induced health benefits are generally accepted. Exercise types are largely divided into aerobic exercises, and these show markedly different adaptations in physiology and metabolism [1–3]. Aerobic exercise has been extensively studied as a mechanism for anti-obesity effects, including improved insulin sensitivity. Aerobic exercise training improves cardio-respiratory capacity, which mobilizes energy using a lot of oxygen, and is recognized as the most effective type of exercise to improve obesity without increasing skeletal muscle mass and strength [3, 4]. It is well established that aerobic exercise increases glucose transporter4 (GLUT4) expression in skeletal muscles by activating AMP-activated protein kinase (AMPK) and Ca^2+^/calmodulin-dependent protein kinase II (CAMKII), which are increased by skeletal muscle contraction and energy depletion [5, 6]. It has been extensively researched as a model mechanism for anti-obesity effect, including improved insulin sensitivity by aerobic exercise training.

Conversely, resistance exercise has been recognized as increasing muscle mass and muscle strength rather than improving cardiorespiratory capacity and obesity metabolism. However, according to a recent studies, resistance training is not limited to simply increasing muscle mass and strength but is also an important type of exercise for improving obesity and metabolic diseases via modulation of metabolic-related signaling transduction [7–10]. Therefore, studies on the physiological and molecular mechanisms of resistance exercise to improve systemic metabolism, including skeletal muscle, liver, and adipose tissue metabolism, are being conducted. This is because skeletal muscle makes up around 40% of the total body weight and is responsible for storing and disposing of glucose and triglycerides (TGs) [8]. Glucose uptake and glycogen storage capacity, in particular, are critical for maintaining and enhancing exercise capacity and daily life.

Recent studies have reported that glucose plays an important role in regulating protein synthesis in skeletal muscles [8, 11–13]. These claims are based on multiple studies which revealed that insulin, which is responsible for glucose disposal, regulates skeletal muscle protein synthesis via several signaling cascades (including phosphoinositide 3-kinase (PI3-K), protein kinase B (PKB) and tuberous sclerosis proteins 1/2 (TSC1/2)-mammalian target of rapamycin (mTOR) complex) [14–16]. These findings imply that insulin sensitivity in skeletal muscle is a critical regulator of maintaining muscle volume and enhancing muscular performance by increasing skeletal muscle protein synthesis.

We were interested in the physiological mechanism of exercise training to cope with metabolic stress. A recent study found that the effects of exercise persisted even after 8 weeks of aerobic exercise training [17]. Even after administrating of a high-fat diet (HFD) for 1 week after discontinuation of exercise, mice that had acclimated to aerobic training through voluntary wheel running for 8 weeks had considerably reduced body weight gain and adipose tissue weight. Furthermore, exercise training counteracted insulin resistance better in response to HFD.

To further explain the findings of our previous studies and the known metabolic effects of resistance training, particularly the preventive effects, we investigated whether the effects of ladder-climbing exercise training counteracted HFD-induced metabolic stress in mice. In addition, we examined the correlation between the improvement in skeletal muscle function and body weight, glucose tolerance and adipose tissue mass through resistance training.

## Results

### Effect of resistance exercise training on skeletal muscle mass and function in mice

**Fig. 1:**
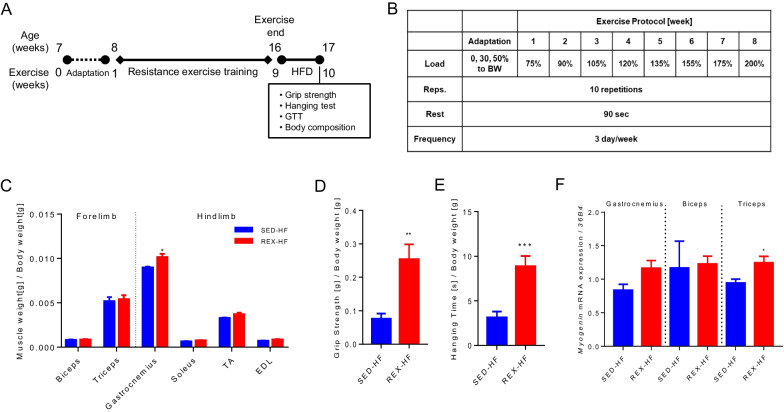
Effects of resistance exercise training on the skeletal muscle mass and function in mice. **A** Experimental scheme of resistance exercise training and HFD. **B** Mice resistance climbing exercise protocol. **C** Skeletal muscle weight (g) to body weight (g) ratio upon sacrifice, n = 4 for each group. **D** Fore-limb grip strength was measured, SED-HF; n = 6, REX-HF; n = 9. **E** The length of time until the mice fell from the hanging wire was recorded, n = 8 for all group. **F** qPCR analysis of myogenin gene expression in skeletal muscles, n = 4. Values are presented as mean ± SEM. Significant differences were determined using an unpaired two-tailed Student’s *t*-test. *p < 0.05

Figure 1A shows the experimental design and work flow, while the resistance ladder-climbing exercise training protocol is shown in Fig. 1B. As shown in Fig. 1C, resistance exercise training did not increase skeletal muscle tissues weight in the forelimbs (biceps and triceps), soleus, tibialis anterior (TA) and extensor digitorum longus (EDL). However, gastrocnemius muscle weight was significantly increased in the resistance exercise-trained group. Forelimb grip strength and hanging time were increased considerably in resistance-trained mice, despite exposure to HFD for 1 week after exercise discontinuation (Fig. 1D and E). The expression of the *myogenin* gene, a marker of muscle growth and muscle stem cell proliferation, significantly increased in the triceps muscle (Fig. 1F). These results show that climbing exercise-induced enhancement of skeletal muscle strength and skeletal muscle endurance capacities were not blunted by training cessation with HFD induction.

### Resistance exercise-induced improvement of skeletal muscle strength is associated with body weight and visceral fat mass

**Fig. 2:**
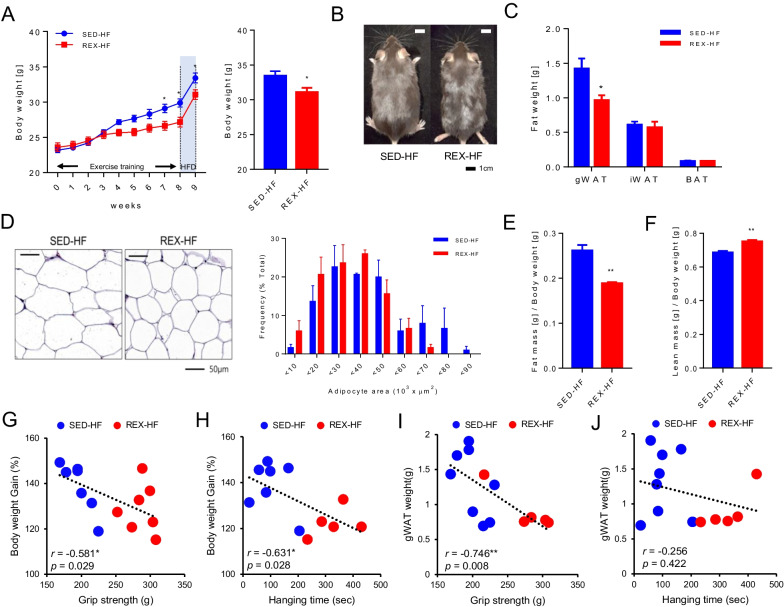
Resistance exercise training improves metabolic phenotype in mice. **A** Weekly body weight (0–9 weeks) (left) and final body weight (right), SED-HF; n = 8, REX-HF; n = 10. **B** The representative image of whole body in each groups (Scale bar = 1 cm); n = 3 for all groups. **C** Fat tissue weight (g), SED-HF; n = 9, REX-HF; n = 10. **D** H&E staining of gWAT and adipocyte size were quantified, n = 3 for each group. **E** Fat mass/Body weight, **F** Lean mass/Body weight was measured, n = 3 for each group. Values are presented as mean ± SEM. Significant differences were determined using an unpaired two-tailed Student’s t-test. *p < 0.05. **G** Correlations between body weight and grip strength. **H** Correlations between body weight and hanging time. **I** Correlations between gWAT weight and grip strength. **J** Correlations between gWAT weight and hanging time. n = 5–8. Statistical differences and r values were determined using Pearson’s correlation coefficient analysis. *p < 0.05, **p < 0.01

Resistance exercise training via ladder-climbing was markedly lowered body weight (Fig. 2A) and body size (Fig. 2B) even after the exercise was discontinued and HFD was induced for 1 week. Furthermore, gonadal white adipose tissue (gWAT) weight (Fig. 2C) and adipocyte size (Fig. 2D) were reduced even after the exercise was discontinued. Body composition data revealed that resistance exercise-trained mice had a significantly lower whole body fat mass/body weight ratio (Fig. 2E) and a higher whole-body lean mass/body weight ratio (Fig. 2F) than sedentary mice. A correlation analysis was performed to explore further the correlation between body weight gain ratio and skeletal muscle function. Significant inverse correlations were observed between the body weight gain ratio and grip strength (Fig. 2G). Hanging time was also significantly inversely correlated with body weight gain (Fig. 2H). However, gWAT weight showed a significant correlation only with grip strength (Fig. 2I), and not with hanging time (Fig. 2J). These results explain the important relationship between improved of skeletal muscle function and the inhibition of body weight gain by resistance training. In addition, these inverse correlations were distinct in the trained and untrained groups despite the training cessation and induction of HFD for 1 week.

### Effect of resistance exercise training on glucose tolerance

**Fig. 3:**
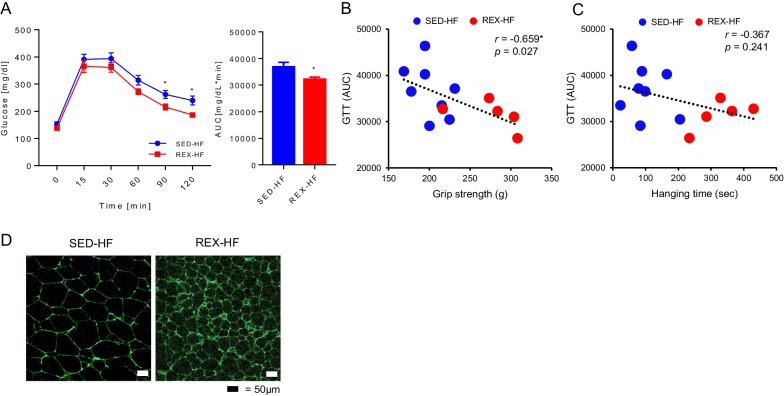
Resistance exercise training improves glucose metabolism in mice. **A** The glucose tolerance test (IPGTT) was measured after glucose injection (1 g/kg/BW), SED-HF; n = 9, REX-HF; n = 8. Values are presented as mean ± SEM. Significant differences were determined using an unpaired two-tailed Student’s t-test. *p < 0.05. **B** Correlations between glucose tolerance and grip strength, n = 5–8. **C** Correlations between glucose tolerance and hanging time. n = 5–8. Statistical differences and r values were determined using Pearson’s correlation coefficient analysis. *p < 0.05. **D** gWAT were visualized using IF with an anti-GLUT4 antibody; n = 3 for all groups

To identify whether the glucose tolerance increased with resistance exercise, we performed a glucose tolerance test (GTT). Resistance-trained mice showed significantly lower glucose levels than untrained mice in response to glucose injection (Fig. 3A). Correlation analysis was performed to explore further the correlation between glucose tolerance and skeletal muscle function. Significant correlations were observed between glucose intolerance and grip strength (Fig. 3B). However, the correlation between glucose tolerance and hanging time was not statistically significant (Fig. 3C). These results suggest that muscle strength is more closely related to glucose homeostasis than muscle endurance. We further assessed the expression of GLUT4, which contributes to glucose homeostasis. As shown Fig. 3D, GLUT4 protein expression was elevated in gWAT of resistance-trained mice. These results suggest that resistance exercise training can regulate glucose homeostasis despite training cessation and HFD induction.

### Resistance exercise-induced metabolic adaptation in skeletal muscle

**Fig. 4:**
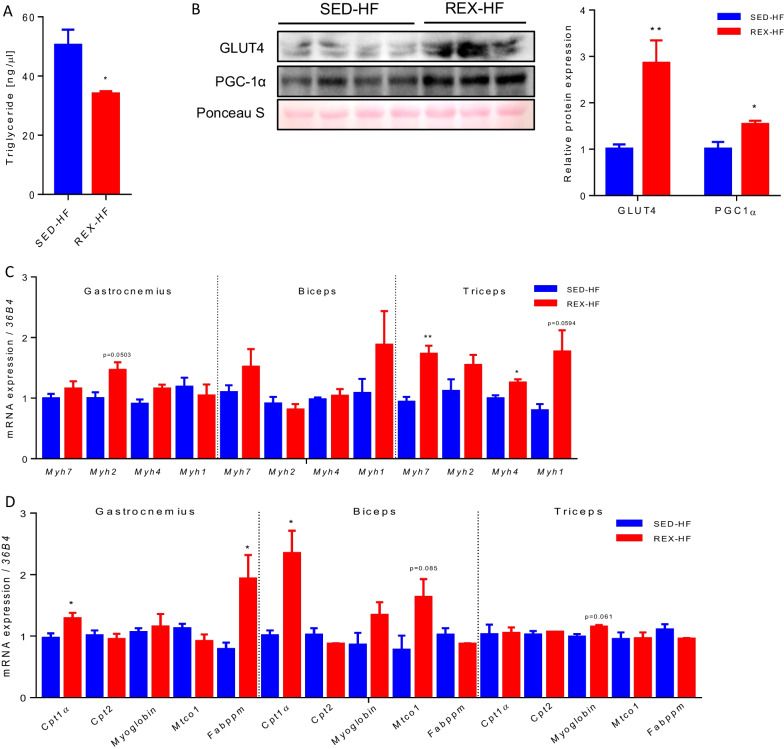
Resistance exercise training improves skeletal muscle metabolism. **A** Gastrocnemius triglyceride (TG) level was measured, n = 5. **B** Western blotting analysis of GLUT4 and PGC-1α protein expression in skeletal muscle, n = 3–4. **C** qPCR analysis of skeletal muscle fiber type gene expression in skeletal muscles, n = 4. **D** Expression of fatty acid oxidation genes in resistance exercise-induced skeletal muscles, n = 4. Values are presented as mean ± SEM. Significant differences were determined using an unpaired two-tailed Student’s *t*-test. *p < 0.05

To evaluate the effects of resistance training on skeletal muscle metabolism, TG levels and glucose and fat oxidation-related markers were measured. Trained mice had significantly lower TG levels in gastrocnemius muscle despite exposure to HFD for 1 week after the cessation of training (Fig. 4A). Furthermore, PGC-1α and GLUT4 protein levels were significantly increased in the gastrocnemius muscle of the trained mice (Fig. 4B). The pattern of *Myh7* and *Myh4* gene expression levels was significantly elevated only in the triceps of resistance exercise-trained mice (Fig. 4C). These results indicate that resistance climbing exercise training specifically stimulates muscle fiber-type gene expression changes only in the triceps. Analysis of fatty acid oxidation-related gene expression in resistance-trained mice revealed a high representation of *Cpt-1α* in both the trained gastrocnemius and biceps muscles (Fig. 4D). In the gastrocnemius muscle, membrane-associated fatty acid-binding protein (FABPpm) mRNA expression levels were also significantly increased. These results suggest that the metabolic response to resistance exercise training differs depending on the type of muscle and is not related to changes in muscle fiber type.

## Discussion

Skeletal muscle mass and strength are recognized as key metabolic indicators and contributors to basic physical function. Therefore, many studies are being conducted to elucidate the physiological and molecular mechanisms by which exercise types affecting the muscles positively affect the whole body metabolism. Physiological adaptations to aerobic and resistance exercises are different. Aerobic exercise primarily enhances mitochondrial production and glucose and fatty acid oxidation in skeletal muscles and increases insulin sensitivity, thereby increasing glucose uptake into the muscle, as is widely documented. Resistance exercise enhances neuromuscular adaptations that increase muscle strength or endurance without significantly changing the oxygen availability. Nevertheless, resistance exercise is an effective form of exercise for improving metabolism. Because resistance exercise induces muscle hypertrophy compared to aerobic exercise, it is advantageous for improving basal metabolic rate and increasing glucose. However, its mechanism is still unknown compared to aerobic exercise.

Our study investigated the preventive effects of resistance exercise on body weight, gonadal fat mass, and glucose tolerance and determined whether the beneficial effects of exercise were associated with skeletal muscle strength and endurance in mice. We observed that grip strength and hanging time were significantly increased in resistance exercise-trained mice, despite exposure to HFD for 1 week after exercise discontinuation. Furthermore, body weight, gonadal fat mass, and adipocyte size were significantly lower in trained mice even with a HFD for 1 week after exercise was discontinued. These results suggest that resistance exercise is a potent regulator of skeletal muscle function and obesity. Although we did not study the molecular mechanisms underlying the preventive effects of resistance training, we analyzed whether resistance training enhanced skeletal muscle function in relation to the body or gonadal fat weight. Surprisingly, muscle strength and endurance had a significant inverse relationship with body weight, and muscle strength had a significant inverse relationship with gonadal fat weight. It was confirmed that this phenomenon could be clearly distinguished depending on whether the training was performed.

Recent human studies have revealed that lower muscle mass and grip strength are associated with metabolic syndrome [9, 18] and a higher risk of developing non-alcoholic fatty liver disease [19]. Another study showed the strongest relationships between resistance exercise and body fat mass and they suggested the importance of combining aerobic exercise with resistance exercise in lowering the risk of obesity [20].

A noteworthy finding from our data was that resistance training significantly increased glucose tolerance despite exercise cessation and HFD. A significant correlation between grip strength and glucose intolerance was also observed. Several prospective human studies have reported a link between relative grip strength and the incidence of diabetes [21, 22]. Skeletal muscle strength is strongly associated with muscle mass, and loss of muscle mass increases insulin resistance [23, 24]. Although mechanical evidence for the relationship between grip strength and glucose tolerance is still lacking, it can be explained by the results that repetitive strength training increases the expression of GLUT4 [25, 26]. Skeletal muscle mass is expected to promote GLUT4 expression and reduce blood glucose levels, since glucose is the major fuel source in skeletal muscle. According to a previous study, GLUT4, which is regulated by transcription factors such as PGC-1α, which regulates mitochondrial biosynthesis in type1 fibers, is also significantly increased in type2 muscle fibers by resistance training [26]. Our data also showed that PGC-1α protein expression and GLUT4 expression increased in gastrocnemius muscle after resistance exercise, despite exposure to HFD for 1 week after exercise was discontinued. In addition, the expression of GLUT4 was significantly increased in gonadal fat. Several studies revealed that GLUT4 is downregulated in adipose tissue of both human and mice with obesity and type2 diabetes [27, 28]. Therefore, the increase in GLUT4 protein expression in gonadal fat by resistance exercise could explain the anti-obesity and anti-diabetes effects of resistance exercise.

## Conclusions

**Fig. 5:**
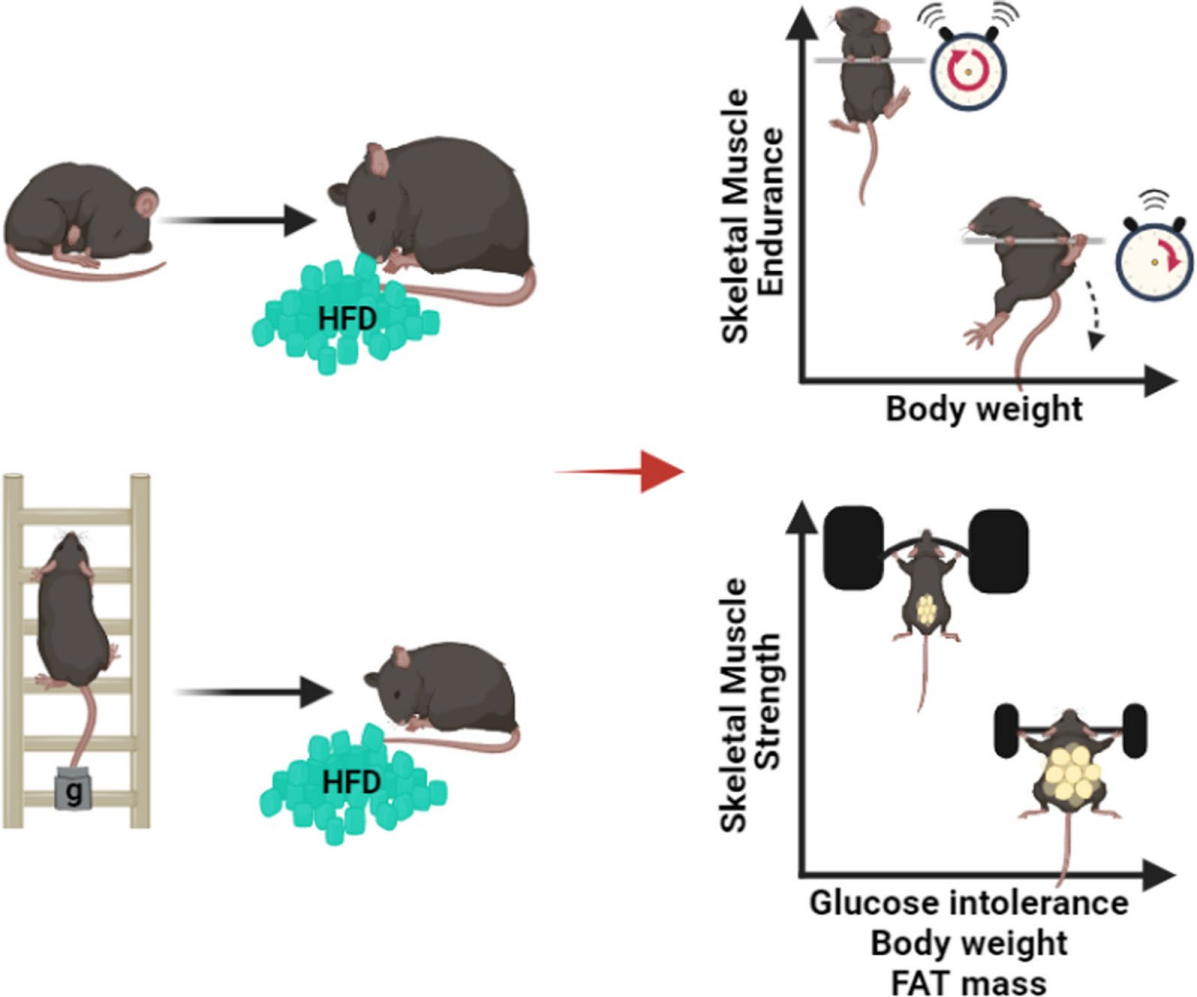
The hypothesis of the relationship between skeletal muscle function and metabolism. Improved skeletal muscle strength by resistance training can dependently lead to glucose tolerance, low body weight and low gonadal fat mass. However, skeletal muscle strength was more correlated with body fat and glucose tolerance than muscle endurance

The mice-climbing resistance exercise improved metabolic parameters and increased muscle strength and endurance. However, muscle strength had a higher correlation with body fat and glucose tolerance than muscle endurance. Furthermore, these effects persisted even under HFD-induced metabolic stress after cessation of exercise. These results suggest that increased and persistent skeletal muscle strength contributes to the lasting effects of exercise on metabolism (Fig. 5). However, further research is needed to determine whether a continuous high-fat diet after cessation of exercise has a lasting protective effect.

## Methods

### Animals

All animal experimental protocols were approved by the Institutional Animal Care and Use Committee (IACUC) of Seoul National University, Seoul, Korea. Permit Number; SNU-200428–2. Male C57BL/6 N mice (22 ± 2 g), aged 8 weeks, were obtained from Daehan Bio Link (DBL) (Seoul, Korea). Mice were housed in a specific pathogen-free barrier facility at a room temperature of 23 ± 1 °C, a relative humidity of 50–60% and a 12 h light/dark cycle. After 1 week of acclimatization, the mice were randomly divided into two groups and maintained on a normal chow diet (NCD, NIH-31, Zeigler, PA, USA). The conditions for one group were the presence of resistance exercise for 8 weeks (8 weeks resistance exercise with NCD + 1 week sedentary with HFD, REX-HF), while those of the other group were the absence of resistance exercise (8 weeks sedentary with NCD + 1 week sedentary with HFD, SED-HF). (n = 8–10 per group). The mice in the exercise group performed regular climbing exercises using a ladder 3 times per week for the entire experimental period (Additional file 1: Figure S1). Training protocol presented in Fig. 1B. The climbing exercise was stopped after 8 weeks and metabolic stress was induced with 1 week of HFD. The body weights of all the mice in both groups were measured weekly. The HFD contained 60% fat, 20% protein, and 20% carbohydrates (in kcal) (D12492, Research Diet, NJ, USA).

### Body composition analysis

Body composition (fat and lean body mass) was analyzed via Nuclear Magnetic Resonance (NMR) methods (Minispec LF-50, Bruker, Germany).

### Glucose tolerance test

Mice that had fasted for 16 h were injected intraperitoneally (IP) with D-glucose (1 g kg^−1^/body weight) (G8270, Sigma-Aldrich, MO, USA). Tail blood was drawn at 15, 30, 60, 90, and 120 min post-injection. Blood glucose was measured using a glucometer (Accu-Check Guide; Roche, Switzerland) at each time point.

### RNA extraction and quantitative real-time PCR

Total RNA was extracted using a TRIzol reagent (A33251, Invitrogen, USA). The RNA concentration and quality were measured at 260/280 nm and 260/230 nm using a Nanodrop-2000 (Thermo Fisher, MA, USA). Next, the cDNA was synthesized from 1 µg of total RNA in the presence of RT premix & master mix (K-2044-B, Bioneer, Korea) at 25 °C for 10 min, 42 °C for 60 min, and 95 °C for 5 min. Real-time qPCR was performed using a Step-One-Plus Real time-PCR System (Applied Biosystems). According to the manufacturer’s instructions, PCR was performed in duplicate using the Sensi-Fast SYBR Green Hi-ROX Kit (BIO-92005, Meridian bioscience, USA). The primer sets for mouse target genes are listed in Additional file 1: Table S1. The primers were purchased from Bioneer. The expression of target genes was normalized to that of *36B4*. All data are expressed relative to each control value.

### Hematoxylin & Eosin (H&E) staining

Fat tissues (gWAT) were weighed and fixed in 4% paraformaldehyde (HP2031, Biosesang, Korea) for 24 h at room temperature. Paraffin-embedded sections were sliced to obtain 3-μm-thick tissue specimens, deparaffinized, and stained with H&E following standard procedures. The cell size was measured using software to calculate the pixels of adipocytes. All slides were analyzed under a Pannoramic Scanner, (3D HISTECH, Hungary) and Image-Pro (Media Cybernetics, USA).

### Immunofluorescence staining

gWATs were directly fixed in 4% paraformaldehyde solution for overnight. Formalin-fixed paraffin-embedded sections (3 μm-thick) were deparaffinized and hydrated, and underwent antigen retrieval using heat-induced epitope retrieval methods. For GLUT4 immunostaining, tissue sections were fixed and permeabilized with 0.02% Triton X-100 in PBS (PBST) for 15 min. Slides were blocked with 5% BSA in PBST for 30 min. Next, the slides were washed once with PBS and probed with anti-GLUT4 antibody (PA5-23052, Invitrogen) at a dilution of 1:500 overnight in 3% BSA in PBS at 4 °C. The slides were then washed three times for 5 min each in 0.05% Tween 20 in PBS, after which they were incubated with Alexa 488-conjugated goat anti-rabbit IgG secondary antibody (A11008, Invitrogen) diluted 1:200 in PBS containing 3% BSA for 30 min at room temperature. Finally, the slides were mounted with a mounting medium (VECTASHIELD, H-1000, USA). Slides were viewed and photographed using an LSM-7700 confocal imaging system (Carl Zeiss, Germany).

### Western blotting

Skeletal muscles were lysed in ice-cold RIPA buffer (50 mM Tris pH 7.5, 150 mM NaCl, 1% Triton X-100, 0.1% SDS, 0.5% sodium deoxycholate, 2 mM EDTA, complete protease inhibitor cocktail, and phosphatase inhibitors). The homogenized tissues were then centrifuged at 13,000 rpm for 15 min at 4 °C. The protein in the supernatant was quantified using a BCA protein assay kit (Thermo Fisher Scientific, USA). Forty micrograms of total protein was resolved on 10–15% SDS-PAGE gels and then transferred to nitrocellulose membranes. All blots were incubated with Ponceau S to ensure equal loading in all lanes. The membranes were blocked with 5% skim milk in TBS with 0.1% Tween20 for 1 h at room temperature to detect the primary antibodies. The membranes were then incubated with GLUT4 (PA5-23052, polyclonal rabbit antibody, 1:2000) (Invitrogen), and PGC-1α (ab188102, polyclonal rabbit antibody, 1:1000) (Abcam, UK) antibodies overnight in 5% BSA in TBS with 0.1% Tween20 at 4 °C. The membranes were then washed three times for 5 min each in TBST, after which they were incubated for 1 h with anti-rabbit or mouse IgG horseradish peroxidase-linked secondary antibody (1:5000) (Cell Signaling, USA). The membranes were then washed as described above, after which enhanced chemiluminescence (ECL) reagents (#1705061, Bio-Rad, USA) were added and analyzed using the ChemiDoc system (Bio-Rad). Target protein levels were normalized against Ponceau staining bands. Blot intensities were quantified using the image J software 1.48 version (NIH, USA).

### TG assay

Mouse muscle TG levels were measured using a TG determination kit (MAK-266, Sigma). All steps were performed according to the manufacturer’s instructions.

### Grip strength

The maximal forelimb muscle strength was measured using a grip strength meter (Bioseb, France). The grip strength of mice was measured according to a protocol described previously [29].

### Hanging test

The endurance strength of the forelimb muscles was evaluated. A mouse was placed on the top of the wire mesh. Subsequently, the wire was turned upside-down. The time taken for the mouse to fall off was recorded.

### Statistical analysis

All data are expressed as mean ± SEM. Differences between the two groups were assessed using a two-tailed Student’s *t*-test. SPSS 25 and Prism 7.0 were used for all statistical analyses. Correlations between metabolic markers and skeletal muscle function were determined using Pearson correlation coefficients. Statistical *p*-values < 0.05 were considered significant. *p < 0.05, ** *p* < 0.01 and ***p < 0.001.

## Supplementary Information

**Additional file 1. Figure S1.** The resistance ladder climbing exercise training of mice. **Table S1.** Primer sequence for qRT-PCR.

## Data Availability

Data supporting the study findings are available from the corresponding authors upon reasonable request.

## Abbreviations

BAT: Brown fat
Cpt1α: Carnitine palmitoyltransferase1α
Cpt2: Carnitine palmitoyltransferase2
Fabppm: Membrane associated fatty acid binding protein
GLUT4: Glucose transporter4
GTT: Glucose tolerance test
gWAT: Gonadal fat
HF: High-fat diet
iWAT: Inguinal fat
Mtco1: Cytochrome c oxidase subunit 1
Myh1: Myosin heavy chain1
Myh2: Myosin heavy chain2
Myh4: Myosin heavy chain4
Myh7: Myosin heavy chain7
PGC-1α: Peroxisome proliferator-activated receptor γ coactivator-1α
REX: Resistance exercise
SED: Sedentary
