# Supplementary material for: Resistance exercise training-induced skeletal muscle strength provides protective effects on high-fat-diet-induced metabolic stress in mice
Source: Lab Anim Res. 2022 Dec 2;38:36. doi: 10.1186/s42826-022-00145-0 (PMC9716768; doi:10.1186/s42826-022-00145-0)
Supplement: Supplementary file 1 — Additional file 1. Figure S1. The resistance ladder climbing exercise training of mice. Table S1. Primer sequence for qRT-PCR. [file 42826_2022_145_MOESM1_ESM.docx]

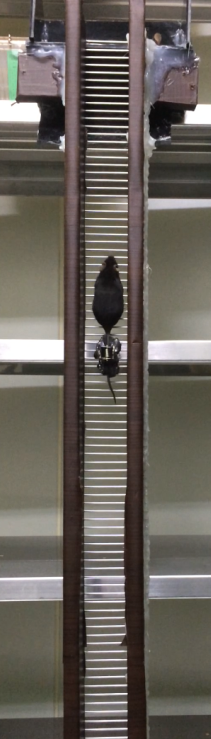


**Figure S1**. The resistance ladder climbing exercise training of mice

|  | Forward (5'->3') | Reverse (5'->3') |
| --- | --- | --- |
| *Myogenin* | CAATGCACTGGAGTTCG (17 mer) | ACGATGGACGTAAGGGAGTG (20 mer) |
| *Myh7* | ACTGTCAACACTAAGAGGGTCA (22 mer) | TTGGATGATTTGATCTTCCAGGG (23 mer) |
| *Myh2* | GCGACTTGAAGTTAGCCCAGGA (22 mer) | CTCGTCCTCAATCTTGCTCTGC (22 mer) |
| *Myh4* | CACCTGGACGATGCTCTCAGA (21mer) | GCTCTTGCTCGGCCACTCT (19 mer) |
| *Myh1* | CGGAGTCAGGTGAATACTCACG (22mer) | GAGCATGAGCTAAGGCACTCT (21mer) |
| *Cpt1α* | TCGATCTCCGCCTGAGCCATGA (22mer) | TCGCGGGGAACACACCAGTGAT (22mer) |
| *Cpt2* | GCTCCGAGGCATTTGTC (17mer) | CATCGCTGCTTCTTTGGT (18mer) |
| *Myoglobin* | CATGGACAGGAAGTCCTCATCG (22mer) | CTGTGAGCACGGTGCAACCATG (22mer) |
| *Mtco1* | CTACTATTCGGAGCCTGAGC (20mer) | GCATGGGCAGTTACGATAAC (20mer) |
| *Fabppm* | GGAAGCAGATAGCGTCCGTG (20mer) | TTCCAGATACCAGCCGAGGA (20mer) |
| *36B4* | GAGGAATCAGATGAGGATATGGGA (24mer) | AAGCAGGCTGACTTGGTTGC (20mer) |

**Table S1**. Primer sequence for qRT-PCR
